# Case of Inflammatory Affection of the Abdomen, Following Parturition, (Puerperal Fever?)

**Published:** 1823-11

**Authors:** H. S. Belcombe


Art. V.—*Case of Inflammatory Affection of the Abdomen, following Parturition, (Puerperal Fever?)* Communicated by H. S. Belcombe, M.D.

Whether the subjoined case is to be considered one of puerperal fever, I will not, amidst the jarring opinions of the present day, attempt to decide. I transmit it, however, to your Journal as a very interesting case; and in the hope that its perusal may not be without effect upon the minds of those who are inclined to follow *peculiar theories.*

To the gentleman who attended with me, Mr. Holland, of Sandbach, I owe many thanks for the energy and promptness with which he seconded my practice.

Mrs. C. aged thirty-four, tall, thin, and very delicate, was safely delivered of a fine child, (her third,) on Thursday, September 11th: the labour was easy and natural, and every thing went on well till Monday evening, when she complained of a sharp pain in the right flank, much increased by inspiration or by pressure. She had had no motion that day; the lochial discharge was regular; the secretion of milk, hitherto copious, had become rather scanty. Her husband abstracted eighteen ounces of blood from the arm, and applied a few leeches; giving her also a dose of calomel and antimony.

The next morning, at eight A. M., I was desired to see her: she then complained of severe pain in the abdomen whenever she moved; the abdomen was a little tumid, and pressure rendered the pain excruciating; the head was easy; the tongue much furred; the countenance anxious; the skin cool; the pulse 80, full and hard; thirst urgent; no evacuation yet procured. Eighteen ounces of blood were immediately abstracted, the smallness of the veins preventing a further flow. She felt sensibly relieved, but not at all faint. Ten grains of calomel and two of antimonial powder were given, and a mixture of magnes. sul. ā infus. sennæ ordered every two hours, till stools were procured. Twenty leeches applied to the abdomen.

At three P. M. the symptoms were aggravated: the pulse quick, sharp, and hurried; the head painful; the countenance very anxious; inspiration producing severe pain; the blood much cupped and buffed: as yet no evacuation. A vein being opened, eighteen ounces of blood were abstracted, when I was again baffled by the smallness of the veins: she, however, felt sensibly relieved, but no faintness. Twenty leeches were applied to the belly, and the bleeding ordered to be promoted by warm poultices. Ol. Terebinth. f. ℥ss. was given, and the mixture as before; an enema was also ordered to be injected every third hour.

Nine P. M.—No amendment. The first dose of the oil was rejected, but the second staid. No evacuation. Belly tumid, and very painful on pressure. The bleeding repeated; twenty leeches again applied; and calomel ℈j. administered.

Four A. M. I met Mr. Holland. The inflammation still hurrying on in full activity; no evacuation. As much blood as we could obtain (about twenty ounces,) was again drawn; the abdomen covered with leeches; and calomel ℈j. pulv. jalap. ʒss. given immediately, and ordered to be repeated every three hours. The enemata continued as before.

Nine A.M.—No progress gained: the pulse as sharp and quick as ever, abdomen just as painful; great exhaustion complained of: no motion. I again had a vein opened, and this time an impression was produced: the pulse fluttered under my finger, and she became exceedingly faint. A large blister was now applied to the abdomen, and the bolus of calomel and jalap repeated.

In three hours after, such alarming depression supervened, attended with so much coldness of the extremities, that I apprehended mortification, and instantly exhibited full doses of ammon. carb. and æther every hour, which relieved her wonderfully. By evening she was much better: had lost all pain of the abdomen; the pulse soft, though yet too quick; countenance tranquil. The bowels at last began to act, and discharged copiously through the night. From this time the recovery was safe and progressive, no untoward symptom, except an obstinate state of the bowels, occurring. The mouth has been slightly, and but slightly, affected. The secretion of milk, which ceased early in the attack, has not returned.

*Newcastle; September 30th,* 1823.

